# Stereotactic radiotherapy for brain metastases: predictive factors of radionecrosis

**DOI:** 10.1186/s40001-023-01178-4

**Published:** 2023-07-13

**Authors:** Benoît Calderon, Léa Vazquez, Mohammed Belkacemi, Nicolas Pourel

**Affiliations:** 1 Institut Sainte Catherine, 250 Chemin Des Baigne-Pieds, 84000 Avignon, France; 2 Nouvelles Technologies, PRECIS, Montpellier, France

**Keywords:** Radionecrosis, Stereotactic radiotherapy, Brain metastases, Predictive factors

## Abstract

**Purpose:**

Stereotactic radiotherapy (SRT) is a highly effective approach and represents the current standard of treatment for patients with limited number of brain metastasis (BM). SRT is generally well tolerated but can sometimes lead to radionecrosis (RN). The aim of this study was to identify predictive factors of radionecrosis related to SRT for brain metastasis.

**Methods:**

This retrospective observational cohort study included patients who underwent SRT in the Institut Sainte Catherine between January 1st, 2017 and December 31st, 2020 for the treatment of brain metastasis from any cancer. Individual data and particularly signs of radionecrosis (clinical, imaging, anatomopathological) were collected from electronic medical records. Radionecrosis was defined as the occurrence on MRI of contrast-enhancing necrotic lesions, surrounded by edema, occurring at least 6 months after SRT and localized within fields of irradiation.

**Results:**

123 patients were included; median age was 66 years. 17 patients (11.8%) developed radionecrosis after a median follow up of 418.5 days [63;1498]. Predictive factors of radionecrosis in multivariate analysis were age under 66 years with a sensitivity of 77% and a specificity of 56%. No other factor as the presence of comorbidities, the number of irradiated metastases, the PTV volume or the volume of irradiated healthy brain were predictive of radionecrosis.

**Conclusion:**

Age at treatment initiation and tumor location seems to be correlated with radionecrosis in patients with brain metastasis treated with SRT. These elements could be useful to adapted radiation therapy.

## Introduction

The true prevalence of brain metastasis is not well known but is probably higher than the available epidemiological estimates worldwide (6–14%) [1–5]. Indeed, the incidence of brain metastases is increasing as cancer therapies improve and patients live longer. Brain metastases derive most frequently from lung cancer (40–50%), breast cancer (15–20%), melanoma (5–20%), renal cancer (5–10%), and cancers of the gastrointestinal tract (5%) [6].

Principal aims of treatment of metastatic disease regardless of its site are to improve quality of life and local control. Radiotherapy, either alone or following resection, remains the mainstay of treatment for brain metastases. Historically, whole-brain radiation therapy (WBRT) was utilized to treat both lesions and the at-risk brain parenchyma for subclinical micro metastatic disease [7]. In 2004, the Radiation therapy Oncology Group (RTOG) 9508 published a randomized trial proving the benefit of stereotactic radiosurgery (SRS) in patient with up to 3 brain metastases [8]. Subsequent trials evaluating the role of WBRT showed a lack of overall survival or functional benefit by adding WBRT to surgery or SRS [9, 10] and even, a lower quality of life [11]. An alternative to SRS is hypofractionated stereotactic radiotherapy (SRT). Several prospective and retrospective studies suggest comparable outcomes for SRS and SRT using doses in the range 24–33 Gy in three fractions [12–14]. Since 2017, SRS is also considered as the standard of care as a less toxic alternative to WBRT, after resection of a brain metastasis [15]. Therefore, to date, SRT represents a highly effective approach and is the current standard of treatment for patients with limited number of brain metastases, after surgery in the operative cavity or as the initial treatment.

Although the risk is minimized compared to SRS, as initial or post-operative treatment, SRT can lead to late toxicities, radionecrosis being the most common [16, 17]. Several studies have described radionecrosis, with or without symptomatic expression. This late side effect occurs in 5–50% of patients and may be associated with neurologic deficits and a significant neurological morbidity [18].

Several predictive factors associated with the development of radionecrosis have recently been researched in some studies, looking at both patient and treatment-related factors. (While a history of) WBRT has been clearly identified as a radionecrosis risk factor, as dose-volume data of normal brain exposed, other predictive factors are still uncertain [8, 19, 20].

Due to the lack of documented data, we conducted this monocentric retrospective analysis in patients receiving SRT for brain metastases to better define bio-clinical predictive markers of radionecrosis.

## Methods

### Patients and study design

**Fig. 1:**
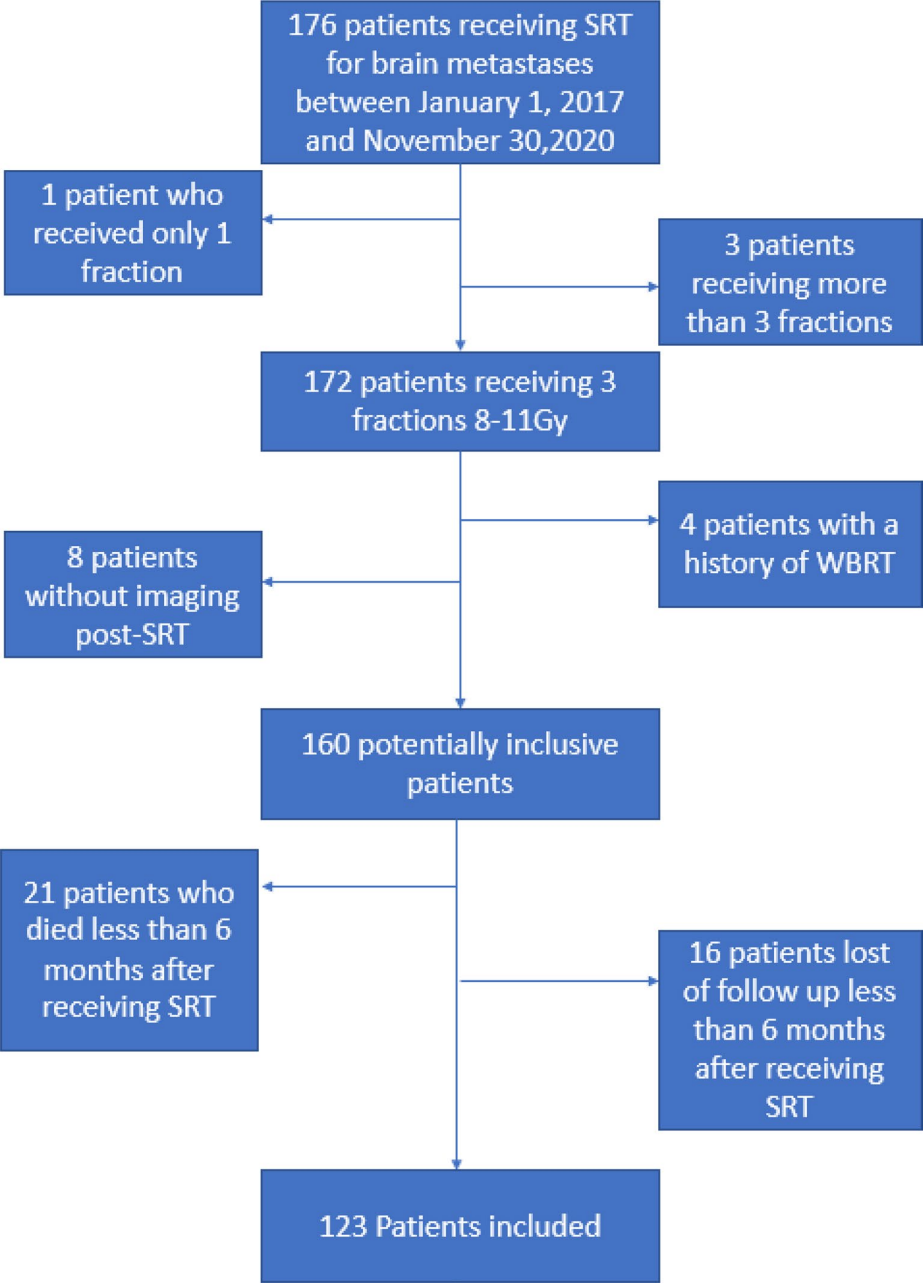
Diagram study

Brain metastases, of any primary cancer treated with SRT as initial treatment or after surgical resection, between January 1st, 2017 and December 31st, 2020 in the Institute Sainte-Catherine were identified via our radiotherapy database. Data from electronical medical report were retrospectively analyzed. To be included in this study, patients need to have received SRT, in 3 fractions of 8–11 Gy, for 1–5 brain metastases. Each patient was followed up for at least 6 months after the last day of SRT for radiation necrosis. Patients without post-SRT imaging, with a history of WBRT, or patients with less than 6 months follow-up were not included (Fig. 1). Data also included Eastern Cooperative Oncology Group (ECOG) index, age, cancer type (primary, histology), comorbidities, number, and localization of brain metastatic sites. Previous and concomitant treatments were also collected. In addition, start and end date of SRT treatment, prescribed dose, PTV size, dose received by the healthy brain, time between diagnosis and start of treatment and time of follow-up were analyzed.

### Radiation therapy

Radiation therapy was delivered with linear accelerator (Truebeam STX, Varian) in stereotactic conditions, using a 5-point thermoforming mask. Treatment scheme were 3 fractions of 8, 9, 10 or 11 Gy, each 48 h. Patient positioning was controlled every day by Cone Beam CT using a 6D table. Treatments used dynamic arc-therapy with 6/18MV photons.

### Endpoints

The main endpoint was to define predictive factors of radionecrosis (RN). Radionecrosis is a common complication after SRT of brain metastases, but no established criteria are available for the diagnosis. The gold standard for this diagnosis is pathological; however, there have been many issues in clinical practice [21, 22]. Thus, comprehensive imaging measure is the most realistic and most frequently used method in the diagnosis of RN [23]. In this retrospective study, the main criteria used for the definition of RN after SRT for brain metastases are detailed in Table 1 [18].

**Table 1:** Criteria used for the definition of RN in our cohort

Criteria	Description of RN
Radiological criteria	The suspected lesions must develop within the radiation fields of a high dose hypofractionated SRT At standard MRI, typical findings include central hypo-signal and peripheral enhancement (contrast-enhancing necrotic lesions) on T1-weighted post-contrast sequence and hypersignal (edema) on T2-weighted sequences An increase of the volume of the tumor followed by shrinkage on serial imaging without anticancer treatment is in favor of RN. RN can also be stable during the follow-up
Pathological criteria	Typical patterns include hypocellular zones of necrosis and fibrinous exudates with degenerative or dystrophic changes in the vasculature, with telangiectasia, hyaline thickening of vessels, fibrinoid necrosis including intravascular thrombosis responsible for an increase of vascular permeability. Dystrophic calcifications can be associated

### Statistical analysis

Descriptive statistics are presented as mean (standard deviation) and median (range) for continuous variables. Discrete variables are reported as count (percentage). The Pearson χ2 test or the Fisher’s exact test, when appropriate, were used to estimate the associations between categorical variables. The two-sided t-test or Wilcoxon rank sum test as appropriate were used for continuous variables. Significance was defined at the p value level below 0.05. Thereafter, a multivariate logistic model was built to analyze the primary end point based on selected parameters from the univariate analysis (the level of significance was set at p-value < 0.20 for selection) and on correlation (Pearson) between these variables. A receiver operating characteristic curve (ROC curve) was performed to know the statistical significance threshold of continuous variables statistically significant on univariate analysis. Estimates of PFS and OS were obtained by the Kaplan–Meier method, and the log-rank test was used to compare differences between survival curves. Statistica (version 13.0) software was used for standard statistical evaluation and SPSS software for ROC curves.

## Results

### Patients (Table 2)

**Table 2:** Patient baseline demographic and clinical characteristics in the radionecrosis population and in the population without radionecrosis

Characteristic	Radionecrosis (n = 17)	No radionecrosis (n = 106)	p value^§^
Age, median [range] years old	60 [44–74]	66 [37–85]	**0.032**
Sex, n (%)			
Male	6 (35.3)	57 (53.8)	**0.16**
Female	11 (64.7)	49 (46.2)	
ECOG performance status, n (%)			
0–1	17 (100)	85 (80.2)	0.22
2–3	0	14 (13.2)	
Unknown	0	7 (6.6)	
Cancer type, n (%)			
ADK	14 (82.4)	86 (81.1)	1
Other	3 (17.6)	20 (18.9)	
Primary site, n (%)			Lung vs Other
Lung	12 (70.6)	68 (64.2)	0.61
Breast	2 (11.8)	16 (15.1)	
Renal	1 (5.9)	7 (6.6)	
Digestive	1 (5.9)	9 (8.5)	
Other	1 (5.9)	6 (5.7)	
Number of treated metastases, n (%)			
1	15(88.2)	64 (60.4)	**0.026**
>1	2 (11.8)	42 (39.6)	
Mutations, n (%)			
Yes	6 (35.9)	32(30.2)	0.67
No	11 (64.7)	74(69.8)	
Treatment history, n(%)			
Chemotherapy	8 (47.1)	68 (64.2)	**0.18**
Stereotactic Radiotherapy	2 (11.8)	9 (8.5)	0.65
Immunotherapy			
Radiotherapy	6 (35.3)	25 (23.6)	0.37
Targeted therapy	6 (35.3)	54 (50.9)	0.23
Hormonotherapy	1 (5.9)	13 (12.3)	0.69
Comorbidities, n (%)			
Yes	3 (17.6)	49 (46.2)	**0.027**
No	14 (82.4)	57 (53.8)	
Treatment during SRT, n (%)			
Chemotherapy	2 (11.8)	10 (9.4)	0.67
Immunotherapy	1(5.9)	8 (7.5)	1
Corticotherapy	15 (88.2)	87 (82.1)	0.47
Levetiracetam	10 (58.8)	51 (48.1)	0.41
SRT spread, n (%)			
≤ 4 days	7 (41.2)	47 (44.3)	0.81
≥ 5 days	10 (58.8)	59 (55.7)	
Post-operative or initial treatment, n(%)			
Initial	10 (58.8)	77(72.6)	0.23
Post-operative	7 (41.2)	25(23.6)	
Both	0	4(3.8)	
Metastases location, n(%)			
Frontal	5 (29.4)	50 (47.7)	**0.17**
Parietal	2 (11.8)	35 (33.0)	**0.08**
Occipital	3 (17.6)	17 (16.0)	1
Temporal	5 (29.4)	19 (17.9)	0.32
Cerebellum	2 (11.8)	28 (26.4)	0.24
Other	3 (17.6)	1 (0.9)	**0.17**
PTV			
Median [range], cm^3^	9.1 [1.3–69.6]	8.25 [1.1–87.4]	0.56
Irradiated healthy brain volume			
Median [range], cm^3^	1321.6 [1191.3–1686.2]	1319.55 [1058.8-1661.6]	0.7
SRT doses (Gy), n(%)			
3 × 11 Gy	10 (58.8)	72 (67.9)	≥ 30 Gy vs ≤ 27 Gy 0.37
3 × 10 Gy	0	2 (1.9)	
3 × 9 Gy	2 (11.8)	6 (5.7)	
3 × 8 Gy	5 (29.4)	26 (24.5)	
SRT-diagnostic BM delay,			
Median [range], days	44 [15–262]	43.5 [13–507]	0.82

^§^ The data were evaluated with χ^2^ test or Fisher’s test when appropriate

¤ The data were evaluated with Wilcoxon rank sum test

Significant results are highlighted in bold

One hundred and twenty-three patients (63 men; 60 women) were included in this retrospective analysis. Median age was 66 years [range 37–86 years old]. Patients had ECOG index 0–4 with only 11.4% (n = 14) known as presenting ECOG > 1. Lung cancers represented 65% (80 patients), breast cancers 14.6% (18 patients), digestive cancers 8.1% (10 patients), and renal cancers 6.5% (8 patients). Among these patients, there were 100 (81.3%) with adenocarcinoma, 9 (7.3%) with squamous cells carcinoma and 14 (11.3%) with another histologic subtype. About the SRT, 70.7% of patients (n = 87) received SRT as initial treatment, 26.0% (n = 32) received SRT as post-operative treatment and 3.3% (n = 4) received both. Most patients (66.6%) received 3 × 11 Gy, 25.2% 3 × 8 Gy (mainly post-operative) and 6.5% 3 × 9 Gy. About previous treatments, 61.8% (n = 76) had received chemotherapy, 25.2% (n = 31) had received immunotherapy, 11.4% (n = 14) had received targeted therapy, 48.8% (n = 60) had received radiation therapy (other than brain) and 8.9% (n = 11) had received intracranial SRT for brain metastases in another site. Concerning comorbidities, 42.3% (n = 52) of patients present at least one comorbidity (as arterial hypertension, diabetes). Most of patients received concomitant corticosteroids (n = 102, 82.9%) and a half, levetiracetam (n = 60, 48.9%). The median time between brain metastases diagnosis and treatment start was 44 days [range 13–507 days].

### Radionecrosis

Seventeen (13.8%) patients presented radionecrosis as previously defined. All of them received an imaging diagnosis and two patients had pathological confirmation. Among the seventeen patients who developed radionecrosis, eight (47%) were symptomatic.

In univariate analysis (Table 2), the factors correlated with radionecrosis were the absence of comorbidities (82.4% vs 53.8%), and the treatment of one metastasis (88% vs 60%); median age at treatment initiation was lower in patients with radionecrosis (60 years old vs 66 years old for patients without radionecrosis). A correlation analysis (Table 3) was conducted between all factors with p value < 0.2 in univariate analysis, allowing us to include only uncorrelated variables in our multivariate model: History of chemotherapy, comorbidities and number of metastases irradiated.

**Table 3:** Significant Pearson’s correlation test

Variables	Coefficient correlation	p value
Sex × comorbidities	− 0.18	0.048
Number of metastases × Frontal metastases	0.33	0.0001
Number of metastasis × Parietal metastases	0.25	0.05

There was no difference among the different cancer types (p = 1), neither between patients who underwent concomitant treatment and the ones who didn’t, neither among the delivering dose (p = 0.37) nor the PTV size (p = 0.56).

As presented in Table 4 logistic regression analysis showed that only parietal or frontal metastases location is predictive factor of occurrence of radionecrosis. The radionecrosis risk is almost 4 times higher when the metastasis is localized in the parietal of frontal region.

**Table 4:** Multivariate analysis of factors associated with radionecrosis

Variables	Final LR model		
	OR	95% CI	p value
History of chemotherapy (Yes vs No)	0.505	0.173–1.45	0.2
Comorbidities (Yes vs No)	0.272	0.0599–0.899	0.093
Number of metastases (1 vs > 1) change for "Parietal or frontal metastases"	0.265	0.119–0.528	**0.01**

*LR* logistic regression; *OR* odds ration; 95% *CI* 95% confidence interval; Significant results are highlighted in bold

**Fig. 2:**
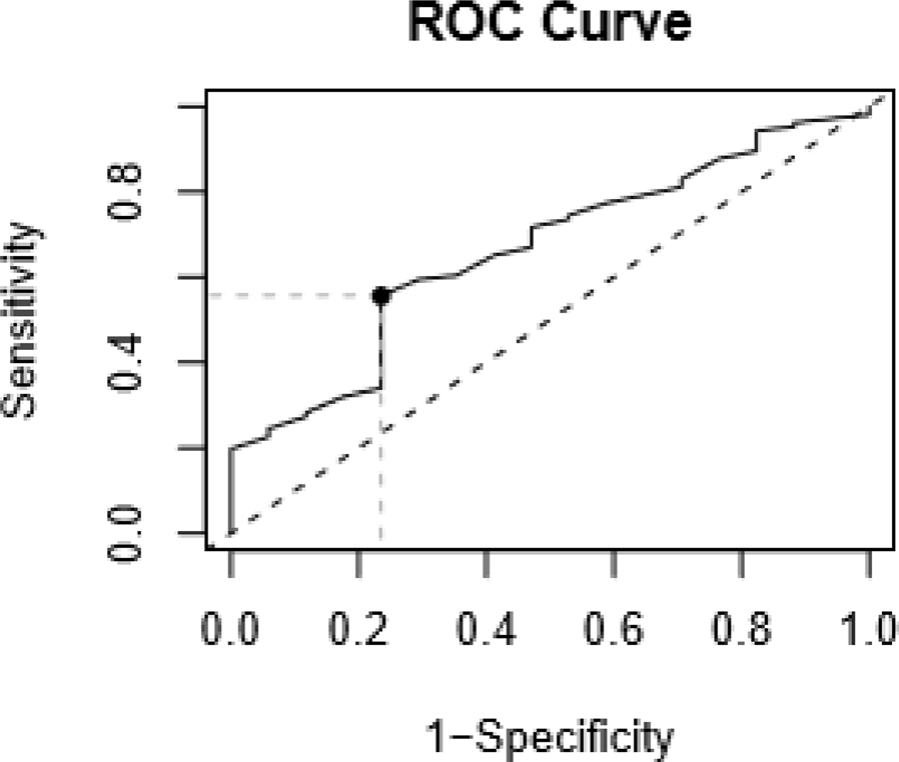
Receiver operating characteristic (ROC) analysis of age at treatment initiation

Concerning age at treatment initiation, a ROC analysis was performed to calculate the threshold value that was predictive of radionecrosis. The cut-off value of 65 years old was determined with a sensitivity of 77% and a specificity of 56% (Fig. 2). In addition, the predictive positive value was 22% and the predictive negative value was 94%.

### Survival

**Fig. 3:**
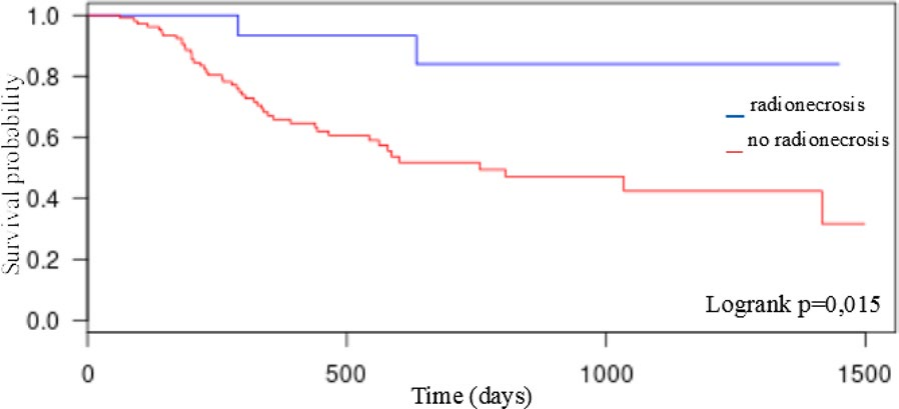
Kaplan–Meier plots of OS: radionecrosis vs no radionecrosis

Median follow-up time was 660 days [101–1449] in the patient’s group who developed radionecrosis and 375.5 days [63;1498] in the other group. Overall survival (OS) analyses included all patients. Median OS of total patient cohort was 1034 [95% CI 0.362;0.625] days. 1-year LC, 1-year and 2-year OS of total patient cohort was 76%, 70% and 56% respectively. Median OS of patient’s group who do not developed radionecrosis was 601 [95% CI 0.415;0.642] days with 46 patients who died at the time of analysis. With 11.8% (n = 2) patients who died at the time of analysis, median OS of patients’ group who developed radionecrosis was not reached. Kaplan–Meier analysis revealed a significantly longer OS for patients who developed radionecrosis (p = 0.015) (Fig. 3).

## Discussion

Few studies have evaluated RN in brain metastasis treated by stereotactic radiation therapy alone, as primary treatment but those published report a rate of RN between 7 and 24% [24–26]. We report a RN rate of 13.8% and a symptomatic RN rate of 6.5%, in agreement with the data reported by the studies already published.

Distinguishing tumor progression and radionecrosis on follow-up imaging is one of the most important challenges after SRT. Both can have similar imaging appearance (enlarging heterogeneously enhancing) complicating treatment decision-making. Ideally, the diagnosis of radionecrosis is established from pathological findings at resection but surgery is associated with the risk of complication and/or neurological aggravation [27]. Nevertheless, imaging showing shrinkage or stability of the lesion without further treatment can lead to radionecrosis diagnostic, whereas active tumor may be demonstrated by sustained growth over multiple follow-up scans [28]. In our study, only 11.8% (n = 2) of patients who were diagnosed with brain radionecrosis by pathology analysis.

Despite several research, the pathophysiological mechanisms involved in brain radiation necrosis is not well understood. From all the studies, clinical or preclinical, published, it appears that radio-induced brain damage is multifactorial. Nevertheless, in a rodent model, vascular injury was observed before radiation necrosis [29]. So, the most likely hypothesis regarding the cause of the development of radionecrosis following SRT is that the direct primary injury to the blood vessels causes the brain parenchymal injury as secondary damage [30]. Both angiogenesis and inflammation may contribute to a synergistic and malignant cycle [31, 32]. Added to neural stem cell damage/oligodendrocytes injury and endothelial cell injury/blood–brain-barrier damage, the intervention of immune mediated mechanisms responsible for the perivascular infiltration on T-lymphocytes and IL-1α, TNF-α, IL-6, and release of cytokine and reactive oxygen species [33].

Our study is the first to have shown that younger age of patients at the time of treatment is a predictive factor of radionecrosis. Indeed, according to our study, patients under 65 years old are statistically more at risk of developing radionecrosis than patients over 65 years old. A previous study on predictive factors of radionecrosis in patients with breast cancer brain metastasis showed a trend to develop more radionecrosis in young patients. In this study, the odds ratio for age did not reach significance, probably due to limited sample size [34]. One hypothesis that may support this finding, may be that immune system, widely involved in the physiopathology radionecrosis process and more efficient among young people, predispose young patients to develop radionecrosis. Nevertheless, RN is a late toxicity, that increases in likelihood as survival increases and younger age seems to be correlated with survival. Indeed, there are conflicting reports about the influence of immunotherapy on rates of radionecrosis: some clinicians are convinced by the synergizing role of immunotherapies on RN rates while others believe its RN rates is revealed by higher patients survival.

The most important risk factors for radionecrosis described with stereotactic radiosurgery are radiation dose, fraction size and subsequent administration of chemotherapy [35]. Furthermore, some studies showed that V10Gy and V12Gy were the most important independent predictors of both symptomatic and asymptomatic radionecrosis [19, 24, 36]. Note that these results all come from SRS studies. Since the dose administration is different in SRT, the analysis of these parameters makes less sense in our study.

Data from SRT studies are uncommon but some studies have demonstrated that V14Gy, V21Gy and V24Gy on healthy brain was a predictive factor of radionecrosis in patient treated for unresected metastases [37]–[39]. Our study including around 30% of patients treated after surgery in the operative cavity, the results are not comparable, the tumor microenvironment being different. Most parameters previously demonstrated as predictive of radionecrosis were not statistically significant in our study. Planning target volume (PTV), healthy brain volume irradiated, comorbidities show no significant differences in multivariate analysis between patients who developed radionecrosis and the others. In our institution, most of the lesions with a diameter > 2.5 cm were treated with a 3 × 8 Gy scheme, while the smallest one was treated with higher dose per fraction. Indeed, the median PTV of tumors treated with 3 × 8 Gy scheme is significantly higher than PTV of tumors treated with 3 × 11 Gy scheme (p < 0.001) which could be consider as a bias and explain the non-signifiance of PTV on our study. However, tumor’s locations are a predictive factor of radionecrosis in our population. Keller et al. have already revealed that location of brain metastases was predictive for radionecrosis after SRT for post-operative resection cavities [40].

In our study, the development of RN may also be related to overall survival, regardless of whether 100% of radionecrosis does not progress locally. Indeed, patients who developed RN had much greater OS than those who did not develop RN, and this result was found regardless of which type of systemic therapy the patient received. Nevertheless, RN is a delayed treatment effect and can occur up to 2 years after SRT. Thus, it is possible that only patients who responded to their systemic therapies will develop RN. However, since the radionecrosis process is immune mediated, it is also possible that patients who develop radionecrosis are patients more sensitive to radiation therapy and systemic treatment. Others monocenter retrospective studies had already shown this trend, however, further prospective study is needed to confirm this finding [41, 42].

Our study has several limitations, including the retrospective design and the absence of pathological RN diagnosis for all patients. However, we used the sequence of several MRIs to confirm the diagnosis. Progressive radionecrosis that could have been mistaken for progression were diagnosed with pathological analysis during local failure surgery. Nevertheless, it is a real-life study with a relatively large number of patients that has identified reliable radionecrosis predictive factor.

In conclusion, this study found that age of patients at the treatment initiation and tumor’s location could predict radionecrosis in patients with brain metastases treated with SRT. Furthermore, patients who developed radionecrosis seems to have a greater overall survival. As the therapeutic window of any new treatment needs to be evaluated for efficacy vs the clinically tolerable safety profile, particularly in the advanced disease setting, these parameters could be easily used for screening a population that could be managed by surgery instead of SRT, irradiation dose reduction or closer monitoring.

## Data Availability

The datasets used and/or analyzed during the current study are available from the corresponding author on reasonable request.

## Abbreviations

SRT: Stereotactic radiotherapy
BM: Brain metastasis
RN: Radionecrosis
MRI: Magnetic resonance imaging
WBRT: Whole-brain radiation therapy
RTOG: Radiation Therapy Oncology Group
SRS: Stereotactic radiosurgery
ECOG: Eastern Cooperative Oncology Group
PTV: Planning target volume
CT: Computed tomography
ROC: Receiver operating curve
OS: Overall survival
